# Preparing medical laboratories in low- and middle-income countries for a sustainable future

**DOI:** 10.4102/ajlm.v14i1.2734

**Published:** 2025-09-16

**Authors:** Tony Badrick, John Anetor

**Affiliations:** 1 Royal College of Pathologists of Australasia Quality Assurance Program, New South Wales, Australia; 2 Department of Chemical Pathology, College of Medicine, University of Ibadan, Ibadan, Nigeria

## Introduction

Hospitals and healthcare facilities generate significant waste and environmental impact.^1^ These effects include generating large quantities of solid and liquid waste, using hazardous materials, consuming large amounts of energy and water, and producing large quantities of wastewater and greenhouse gases.^2^ Because of the specialised nature of the equipment used and the need to tightly control temperature and humidity in medical laboratories, they typically use three to six times more energy per unit surface area. They also consume 60% – 80% more energy than a typical office building.^3^ Medical laboratories, in addition, generate large quantities of chemical (toxic) wastes, clinical glassware, culture plates, stock cultures, and highly infectious wastes in massive amounts. Of particular concern is radioactive wastes, hazardous and infectious wastes that require packaging, handling, and special treatment methods.^4^ Inappropriate management of these dangerous wastes can result in ill health in staff in healthcare facilities, patients, waste collectors, and the public at large, because of exposure to these health risks.^5^

Within the laboratory, there is a high level of understanding of the risks, especially in higher-income countries. In contrast, in other laboratories, particularly in the low- and medium-income countries,^6^ understanding of the broader risks to the community of laboratory waste is less well appreciated. While guidelines and educational campaigns are available, there is still only a rudimentary understanding of how sustainability guidelines can apply to diagnostics laboratories and equipment.^7,8,9,10^

In the low-and middle-income countries,^6^ regulations and guidelines are being used to reduce the industry’s environmental impact, but there is still a poor understanding of how sustainability guidelines can apply to medical laboratories in most developing countries especially in Africa. Modifying existing products to meet sustainability requirements is rarely an option, leaving new product development as the only alternative long-term approach to an immediate problem.^2^ The problems are multiplied manyfold in low- and middle-income countries where there are numerous issues, including scarcity of financial resources, priority of the country, natural calamities, and conflict between governments and trade unions. Organisational profit-making schemes with sustainable technologies, corruption at various levels, lack of interest, and efforts to incorporate sustainable development goals are other major obstacles to enabling sustainability in laboratory medicine in the low- and middle-income countries.^11,12^

Recognising these issues, the International Federation of Clinical Chemistry and Laboratory Medicine (IFCC) responded by creating a Task Force on the Environmental Impact of Laboratory Medicine. The IFCC has been active in this area, with the formation in 2012 of an earlier ad hoc IFCC Panel on the Environmental Responsibility of Clinical Laboratories.^8^ The IFCC panel produced a set of proposals for mitigating the environmental impact of clinical laboratories.

The new IFCC Task Force on the Environmental Impact of Laboratory Medicine was created with the following goals, and an intended focus on developing countries:

- ‘Identify existing peer-reviewed, high-quality publications that describe the impact of laboratory medicine operations on the environment and actions that laboratories and manufacturers can take to reduce the negative impact.
- Develop recommendations and practice guidelines that laboratories in both developed and developing countries can implement to reduce the environmental impact of laboratory operations without compromising the quality of services provided to patients.
- Develop a plan to share information and educate IFCC national societies and corporate members.
- Identify existing peer-reviewed, high-quality publications that describe the laboratory testing that can be performed to measure both levels and biological effects of toxic environmental chemicals in human biological material, including studies to demonstrate the concentrations of chemicals found in human biological tissues (human biomonitoring).’
- As required, the Task Force will establish a formal collaborative link with the European Federation of laboratory Medicine Task Force on Green Labs and other IFCC groups and organisations involved in related activities.^13^

## Planning for low and medium-income country laboratories to embrace sustainability in laboratory medicine

Understanding the cultural, organisational and behavioural changes are necessary to implement long-term and sustainable changes. It requires strong and unwavering leadership with a well-defined and documented plan. It would also require external support from trusted mentors and internal champions who understand the importance of the task ahead. It may also be important to stress that there is a long-term economic benefit in implementing sustainable laboratory medicine practice.

The plan would include the following components:^8^

- Establish and maintain procedures that specify environmental objectives and targets and direct efforts towards continual improvement. These need to be realistic, given the resource constraints that many laboratories face in these countries.
- Minimise appliance energy and water consumption. There are often relatively easy ways to reduce energy usage, such as simply switching equipment or lights off when they are not in use (https://greenlabs.eflm.eu/documents/Presentation_Energy.pdf).
- Reduce waste by reducing the use of resources and embrace the reuse or recycling of materials (https://greenlabs.eflm.eu/documents/Presentation_Waste.pdf).
- Review environmental practices at least annually. This ensures that the plan is current and updated with new projects added as appropriate.
- Engage in advocacy by encouraging customers, suppliers and other stakeholders to mirror the organisation’s commitment to environmental responsibility.
- Green purchasing policy purchasing should be incorporated into purchase contracts ahead of final purchase of major equipment and consumables.
- Environmental managers may be appointed by each laboratory to take charge of toxic chemical use and appropriate disposal as well as possible recycling
- (https://ifcc.org/executive-board-and-council/eb-task-forces/task-force-environmental-impact-of-laboratory-medicine-tf-eilm/).

## Implementation of sustainable practices and economic benefits

Many universities and institutes across the globe have implemented sustainability projects. One of the most prominent ones is the University of Groningen, Faculty of Science and Engineering, University of Groningen, the Netherlands.^14,15^ The integration of sustainable practices in clinical laboratories as part of a global scientific community embracing sustainability in science is important for mitigating environmental impact promoting resource conservation, and in ensuring the long-term viability of healthcare systems. Collaboration and innovation in laboratories can lead the way toward a healthier and more sustainable environment.

The economic benefit of sustainable laboratory practice is evident from the examination of standaedised data from selected hospitals and other institutions that have implemented sustainable programmes. The data revealed that economic benefits of intervention exceeded $5.4 billion dollars over a 5-year period and $15 billion dollars over a 10-year period with an estimated gross net savings of over $15.2 billion dollars in the long run. Returns were almost tripled over 10 years.^16,17^

Briefly, the barriers to embracing sustainable practices are erroneous phobias about initial investment.^16^ These can be addressed by innovative and committed leadership, as well as education and persuasion, as elegantly demonstrated by Freese et al.^15^

Appropriate leadership will lead to policy formulation and regulation that will guide organisations, also elegantly discussed by Freese and his colleagues.^15^ Sustainability has enormous scientific, social and economic benefits that can only be achieved or embraced by persuasion at this time. Regulations that are enforceable may follow.

In an opinion paper such as this, it is not possible to include too many details, but again, the paper by Freese et al.,^15^ and several others, have comprehensively addressed aspects of the technology.^18^

It is, therefore, a public health and laboratory medicine priority in many low- and medium-income countries, especially those in Africa, to put measures in place to ensure sustainable laboratory medicine practices. In the science of toxicology, there is a well-recognised axiom in pollution science and exposure control: ‘poison one part of the globe and the whole world is poisoned’.^19^ Pollution has no respect for international boundaries; this is why international partners must join hands with low- and medium-income countries in enthroning sustainable laboratory medicine practices. The medical laboratory community must form a partnership with low- and medium-income counties to eradicate laboratory medicine-associated adverse environmental impacts and ensure green laboratory medicine practice for all for the future.
